# Charge-Induced Polarization in Dielectric Particle Systems: A Geometry-Dependent Effect

**DOI:** 10.1021/acs.jctc.5c00544

**Published:** 2025-06-12

**Authors:** Eric B. Lindgren

**Affiliations:** Departamento de Físico-Química, Instituto de Química, 28110 Universidade Federal Fluminense, 24020-141 Niterói, Rio de Janeiro, Brazil

## Abstract

Electrostatic interactions in systems composed of finite-sized dielectric materials extend well beyond simple point-charge approximations, particularly when many-body polarization effects become significant. This study shows that asymmetries in the size or net charge of spherical particles can trigger nontrivial phenomena, including like-charge attraction and intricate force balances involving neutral species. Through a rigorous boundary-integral framework, it is substantiated that induced surface charges propagate through iterative cascades, reflecting the full many-body, nonadditive character of polarization. Significantly, a geometry-based cutoff is adopted to discriminate whether long-range interactions can be approximated by monopoles, thereby retaining near-field multipole couplings without forfeiting computational efficiency. This approach provides significant computational gains without compromising the rigor of many-body treatment, underscoring the critical interplay between geometric factors—specifically, particle size (and its associated curvature) and interparticle separation—in determining local field intensities, which often exceed conventional Coulombic predictions. The findings can illuminate pathways for understanding and designing advanced materials and self-assembled architectures in which dielectric polarization governs or contributes to emergent behavior.

## Graphical abstract

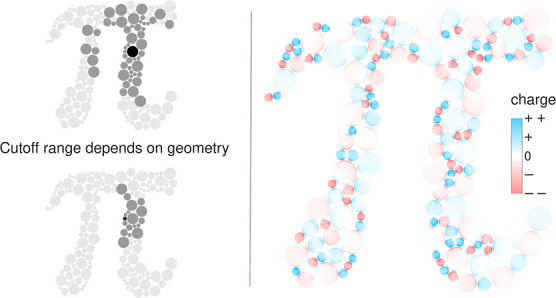

## Introduction

Electrostatic interactions underpin the structure and behavior of matter, governing interatomic and intermolecular forces across all scales. At the most elementary level, these interactions originate from the Coulombic forces between the charged constituents of atoms—protons and electrons—establishing the foundation for intermolecular phenomena.^1^ Ionic interactions, dipole–dipole forces, and higher-order multipole interactions emerge directly from these classical electrostatic principles. However, the electrostatic landscape extends beyond fixed charge distributions as all matter exhibits an intrinsic capacity to be polarized.^2^ The ability of electrons to shift relative to nuclei in response to external electric fields enables the induction of dipole moments, while higher-order multipoles can also contribute, particularly in asymmetric geometries or in the presence of strong, nonuniform fields.^3^

Unlike Coulombic interactions, which arise from pairwise summations of discrete charges, polarization in a many-body system, such as an ensemble of dielectric particles, results from the collective interplay of induced multipolar moments. The electric field that polarizes a given particle is not determined solely by the fixed charges of neighboring particles but also by the contributions from the full spectrum of induced moments (including dipolar, quadrupolar, and higher-order terms), whose strengths and orientations depend on their polarizability and local environment.^2,4^ This mutual dependence creates a feedback loop: the polarization induced in one particle alters the local field experienced by others, thereby modifying their multipolar responses and, in turn, influencing the original field. Consequently, the response of a particle to an external field cannot simply be decomposed into independent additive contributions from individual neighbors, as each effect is intrinsically linked to the collective state of the entire system. This interdependence makes polarization a fundamentally nonadditive effect.^5^

The critical role of polarization is evident across diverse processes governed by electrostatic interactions. When particles are suspended in a medium, the local dielectric environment modulates their effective interactions. Induced multipole moments—predominantly dipolar but also including significant higher-order contributions—can lead to either attractive or repulsive forces, depending on factors such as the relative polarizability of the particles, the dielectric properties of the surrounding medium, and the spatial distribution of external or intrinsic electric fields.^2,6^ The medium thus plays a crucial role in screening effects and modifying electrostatic interactions, making polarization particularly significant in its absence. In vacuum (free space), where no external medium attenuates the fields, polarization forces—arising solely from the intrinsic polarizability of the particles and the direct influence of their mutual fields—can dominate the interaction. These effects are particularly significant in gas-phase environments and astrophysical contexts, where charged or polarizable particles interact with virtually no dielectric screening. Examples include the behavior of aerosols^7–9^ and dust particles.^10–12^

Across length scales, the interplay of electrostatic forces and polarization manifests uniquely, shaping interactions from the atomic level to the macroscopic level. At the molecular scale, polarization influences bond strengths,^13,14^ molecular recognition,^15,16^ and reaction mechanisms^17,18^ by modifying local electrostatic environments. In mesoscopic systems, such as colloidal suspensions and nanoparticle assemblies, induced charges may dictate stability,^19–22^ phase behavior,^23–25^ aggregation, and self-assembly.^20,21,24,26–28^ It is worth noting, however, that polarization rarely operates in isolation; it frequently works in concert with other forces (e.g., van der Waals attractions, steric hindrance, depletion interactions), resulting in synergistic or competing effects that can strongly influence the resulting structures and their properties.^2^ On macroscopic scales, polarization underlies charge storage in capacitors,^29,30^ enables tunable interactions in electrorheological fluids,^31,32^ and influences electrostatic adhesion in robotics and soft actuators,^33,34^ all of which similarly benefit from the interplay between induced charges, besides additional mechanical or electrostatic forces present in these systems.

Interactions involving point particles (e.g., point charges) fundamentally differ from those involving finite particles due to the latter’s polarizable volume. A point particle is a mathematical construct, representing a charge or mass concentrated at a single point in space, with zero spatial extent and, consequently, zero volume.^35^ As a result, in the absence of relativistic and magnetic effects, interactions between point charges are always governed by Coulomb’s law, which accounts only for direct charge–charge forces. By definition, point particles cannot undergo polarization as there is no spatial distribution of charge that can be distorted by an external field. Indeed, quantum mechanics predicts that the spatial probability density of detecting a fundamental particle (e.g., an electron) is delocalized according to its wave function, leading to statistically averaged measurements that mimic a distributed charge. However, the particle is treated as a point-like entity in the quantum field theory. These quantum effects, such as wave function-driven delocalization, are typically negligible in macroscopic systems due to decoherence and the dominance of classical physics at larger scales.^36,37^ Since point charges lack internal structure, the force between them depends only on their separation distance, following Coulomb’s inverse-square law: it approaches zero as their separation tends to infinity and diverges as their coordinates converge. In contrast, finite particles possess a spatial extent and, consequently, a polarizable volume, meaning that their internal charge distributions can shift in response to external electric fields. This polarization effect introduces an additional interaction term that depends not only on the distance between the particles but also on their size and shape.^2^

When two polarizable particles interact, particularly if both carry a net charge, the degree to which polarization influences the interaction is strongly dependent on their separation. If the distance between them far exceeds their characteristic sizes, the internal displacement of charge (typically manifested as a small induced dipole moment) remains minimal. In this case, regardless of their orientation, the effect of polarization on the net interaction is weak. However, as the particles approach each other, the separation between the induced positive and negative regions may become comparable to the interparticle distance. When this occurs, the polarization interaction becomes significant, adding a distance-dependent force beyond the simple Coulombic term.^2^ Thus, the geometric configuration, namely, the size of the particles and their spatial arrangement, evidently plays a crucial role in determining the significance of polarization effects.^38,39^ Unlike point charges, for which the interaction is purely a function of distance, finite, polarizable particles exhibit a more complex interplay of charge distribution and electrostatic forces, making polarization an inherently many-body phenomenon that depends on the system geometry.

This work explores the significance of polarization effects in the interactions between spherical dielectric particles, examining how factors such as particle size, charge, dielectric constant, and separation distance contribute to polarization and ultimately influence the overall interaction. The study demonstrates how polarization can enhance attraction, weaken repulsion, and even enable attractive interactions between like-charged particles. A range of system configurations is considered, beginning with fundamental two-body interactions and extending to many-body scenarios, to systematically illustrate the role of polarization in interactions involving both charged and neutral dielectric particles. Particular emphasis is placed on the role of geometry, showing that the significance of polarization depends critically on system configuration, dictating when it must be taken into account and when it can be safely neglected. To address this, the adoption of a geometry-based cutoff parameter is proposed to establish the conditions under which polarization effects should be included, ensuring computational efficiency while maintaining control over accuracy loss. The application of this parameter is demonstrated for interactions between charged dielectric spheres, although its formulation can be readily extended to analogous systems where the interacting particles may be treated or approximated as spherical entities. This framework has broad relevance to the modeling of natural phenomena (e.g., atmospheric aerosols) and engineered systems (e.g., electrostatically driven self-assembly), offering insights into collective interactions in which polarization and geometric arrangement critically influence emergent behavior.

## Model Overview

Recent advances in the computation of electrostatic forces between charged particles have introduced alternatives to traditional image charge methods, which often exhibit convergence issues at short separations.^40^ More robust and precise approaches, such as multipole expansions,^41–44^ bispherical coordinate methods,^45,46^ and integral equation formulations,^43,47,48^ now provide computationally stable solutions even for closely interacting dielectric particles. In this work, all calculations were performed using the model developed by Lindgren et al.,^48^ a numerical framework designed for computing electrostatic interactions among multiple dielectric spherical particles. This method is based on a second-kind boundary integral equation discretized via a Galerkin approach, ensuring numerical stability and rapid convergence.^49^

The system described by the model consists of a collection of *M* nonoverlapping spheres Ω_*i*_ (*i* = 1, 2, ..., *M*) in $R^{3}$ , each with a radius *a*_*i*_ and center *x*_*i*_. The surrounding isotropic medium, Ω_0_, is homogeneous, devoid of free charge carriers, and characterized by a relative permittivity (dielectric constant) ε_m_, while each particle Ω_*i*_ has a relative permittivity ε_*i*_. The dielectric distribution in space is described by the piecewise function

$$\varepsilon \left(x\right)=\varepsilon_{m}+\sum_{i\in \left\{1,...,M\right\}}\left(\varepsilon_{i}-\varepsilon_{m}\right)1_{\Omega_{i}}\left(x\right),,x\in R^{3}$$

where $1_{\Omega_{i}}$ is the characteristic function of Ω_*i*_. Each particle carries a surface free charge *q*_*i*_, defined by the isotropic charge density

$$\sigma_{f,i}=\frac{q_{i}}{4\pi a_{i}^{2}},,x\in \Gamma_{i}$$

where Γ_*i*_ = ∂Ω_*i*_ (*i* = 1, 2, ..., *M*) represents the surface (boundary) of each dielectric sphere Ω_*i*_. The global charge density function is given by

$$\sigma_{f}\left(x\right)=\{\begin{array}{ll} \sigma_{f,i} & \mathrm{if}\;x\in \Gamma_{i},\\ 0 & otherwise \end{array}$$

The system is governed by the Laplace equation within each dielectric domain:

1 $$\Delta \Phi \left(x\right)=0,x\in \Omega_{0}$$

2 $$\Delta \Phi \left(x\right)=0,x\in \Omega_{i},\;i=1,\;2,...,M$$

Boundary conditions at the particle surfaces Γ_*i*_ ensure continuity of the electrostatic potential and enforce the discontinuity in the normal component of the displacement field due to surface charge:
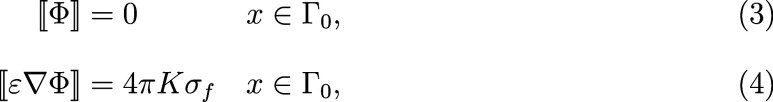
where $\Gamma_{0}=\overset{M}{\underset{i=1}{\cup }}\Gamma_{i}$ . *K* is Coulomb’s constant, and the jump conditions are defined as
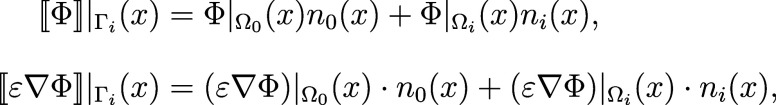
where *n*_*i*_(*x*) and *n*_0_(*x*) are the outward normal vectors to Ω_*i*_ and Ω_0_, respectively.

The potential Φ is solved using an integral equation formulation, discretized via Galerkin projection onto the basis of real spherical harmonics truncated at a chosen degree *N*.^48^ Subsequent analysis has demonstrated that the method exhibits *M*-independent error bounds and maintains reliable accuracy for large-scale systems, ensuring its suitability for extensive simulations.^50^ For two-body interactions, it has been verified that this model yields numerical results identical to the analytical solution of Bichoutskaia et al.,^44^ which employs a rapidly converging multipole expansion based on Legendre polynomials.

It should be noted that a uniform isotropic relative permittivity is assigned to the entire volume of each sphere in the formulation. Spatial variations of ε near boundaries have been documented, most notably by Lekner for noble-gas liquid–vapor interfaces.^51^ In that planar case, the anisotropic interfacial region spans only *t* ≈ 1.3–1.4 atomic diameters (approximately 0.5–0.6 nm for noble gases), altering macroscopic observables by just 1–2%. Similar conclusions have been reached for other dielectric interfaces, where continuum models—whether they employ a uniform bulk ε or a spatially varying diffuse profile—remain accurate down to nanometer scales.^52,53^ For micrometer-scale particles, such as the 10 μm radius spheres treated here, this interfacial shell would occupy <10^–4^ of the radius, so its integrated effect is negligible compared with the large geometry-driven changes produced by many-body polarization, which is often substantial and, in some configurations, can even reverse the force sign. The constant-ε assumption is therefore appropriate and keeps the many-body problem tractable. For nanometer-sized particles, the same approximation should introduce a systematic uncertainty of only a few percent, an inherent limitation of the continuum treatment.

Although the original boundary-integral framework is capable of accounting for a polarizable dielectric medium^48^ and was subsequently extended to include ionic charge carriers through a linearized Poisson–Boltzmann description,^54^ these aspects are deliberately omitted here. Emphasis is placed on how finite-size polarization within the particles can overturn simple Coulombic expectations. Consequently, an idealized vacuum environment is considered in all calculations so that such polarization effects emerge most clearly. In applications requiring a rigorous treatment of an embedding medium or ionic screening, the integral-equation formalism can be reintroduced with the additional components described by corresponding publications.^48,54^

## Results and Discussion

### Two-Body Systems

The most fundamental yet physically insightful case of electrostatic interaction between dielectric particles involves two identical, charged bodies. As illustrated in Figure 1a, a pair of identical dielectric spheres are positioned in close proximity, each sharing the same net (free) surface charge, relative permittivity, and radius. Due to their like charges, they experience a repulsive electrostatic force. Notably, the interaction remains strictly repulsive, irrespective of the constituent relative permittivities. Within this symmetric system, polarization effects modulate solely the magnitude of the repulsive force. Accordingly, as the relative permittivities of the spheres increase (with ε_1_ = ε_2_, ε_m_ = 1), the magnitude difference between opposing bound charges amplifies, driving a progressively stronger attenuation of the repulsive force.

**Figure 1:**
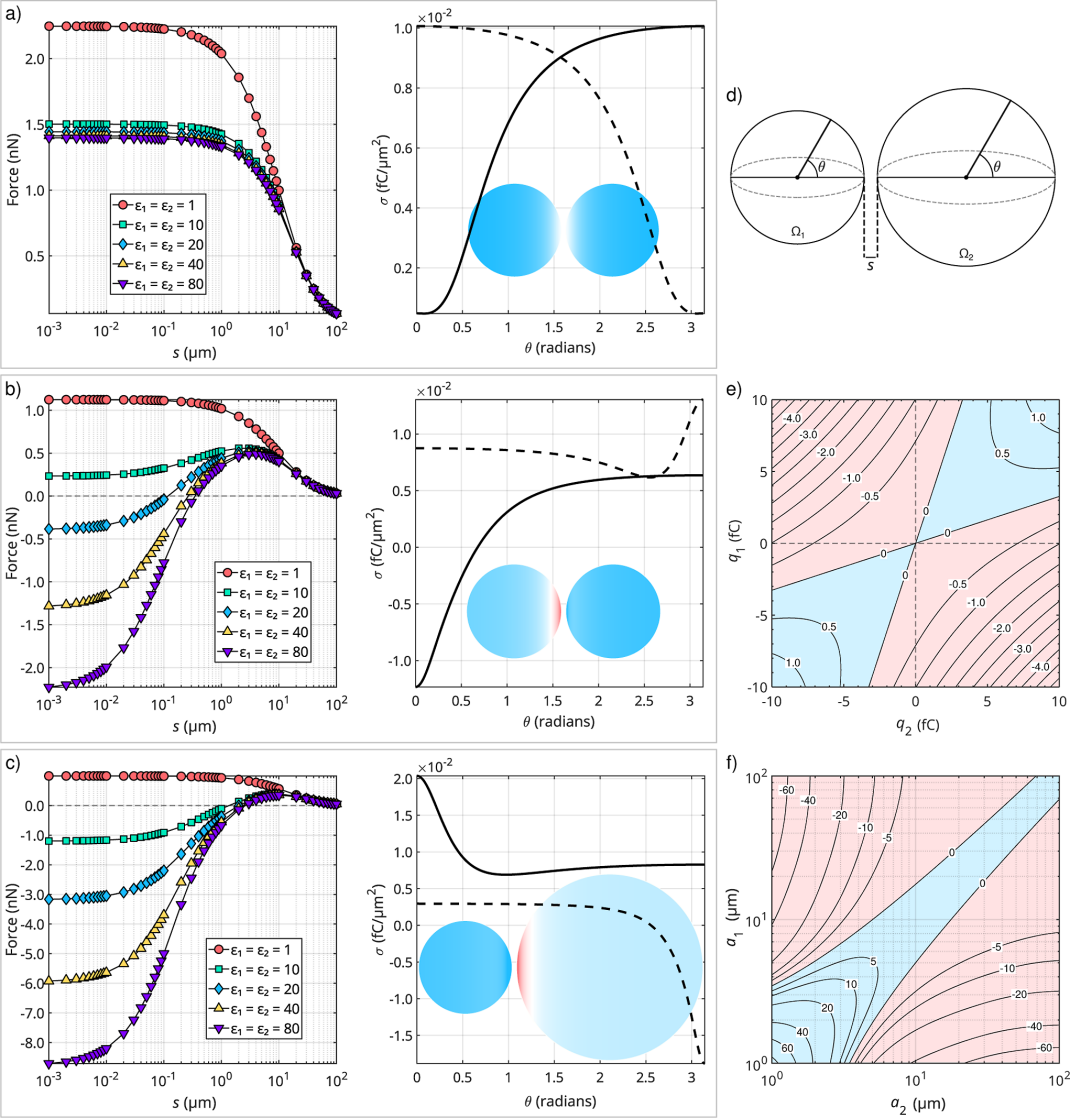
Two-sphere interactions. (a–c) Electrostatic force as a function of the surface-to-surface separation, *s* (plots on the left), and the surface charge density, σ, as a function of the polar angle, θ (plots on the right), for two interacting spheres, Ω_1_ and Ω_2_. Force curves are presented for selected values of ε_1_ = ε_2_, as indicated. The surface charge distribution is computed at *s* = 1 μm for ε_1_ = ε_2_ = 20, with the solid curve representing Ω_1_, and the dashed curve representing Ω_2_. Blue hues on the spheres indicate positive charge, red hues indicate negative charge, and white denotes electrically neutral areas. (a) Symmetric configuration, where *a*_1_ = *a*_2_ = 10 μm and *q*_1_ = *q*_2_ = 10 fC. (b) Configuration with asymmetry in charge, where *a*_1_ = *a*_2_ = 10 μm, *q*_1_ = 5 fC, and *q*_2_ = 10 fC. (c) Configuration with asymmetry in size, where *a*_1_ = 10 μm, *a*_2_ = 20 μm, and *q*_1_ = *q*_2_ = 10 fC. (d) Schematic of the geometry, denoting the quantities varied in the plots. (e) Contour plot of the electrostatic force (nN) as a function of *q*_1_ and *q*_2_, with *a*_1_ = *a*_2_ = 10 μm; regions of repulsive regime are rendered in a light-blue tone, whereas those corresponding to an attractive regime are displayed in a light-red tone. (f) Contour plot of the electrostatic force (nN) as a function of *a*_1_ and *a*_2_, with *q*_1_ = *q*_2_ = 10 fC. In all cases, ε_m_ = 1 and *N* = 15, except for the surface charge density graph, where *N* = 35. For the contour plots, ε_1_ = ε_2_ = 20 and *s* = 1 μm.

Due to exact congruence in both the physical and geometrical parameters of the system, the equilibrium distribution of induced surface charges must also reflect this symmetry. Specifically, interchanging the two particles or reflecting them across the bisecting plane leaves the overall configuration unchanged. As a direct consequence, the solution to the electrostatic problem yields induced charge distributions that are exact mirror images of the opposing hemispheres. This symmetric charge arrangement arises naturally from the governing Laplace equation, which, under symmetric boundary conditions, admits a unique solution that preserves the structural invariance of the system.^55^ As long as the underlying physical assumptions—linearity, isotropy, and perfect symmetry—hold, no alternative equilibrium configuration is permissible.^4^

Within each dielectric domain, the electrostatic potential Φ satisfies Laplace’s equation and adheres to well-defined boundary conditions at the interfaces between media. Since these conditions are symmetric for two identical spheres, the uniqueness theorem ensures that the resulting solution Φ­(*x*) preserves this invariance.^55^ Consequently, the induced charge distributions on the two spheres remain perfectly mirrored (Figure 1a). This balanced arrangement in induced charge distributions is not an artifact of the model but a direct consequence of Maxwell’s equations.^4^ In reality, experimental imperfections—such as minor variations in geometry, material properties, or external influences—may break this ideal state, leading to small deviations in the charge distribution. However, the fundamental electrostatic principle remains unchanged: under ideal conditions, the system enforces a perfectly symmetric equilibrium.

When the ideal balance of the system is disrupted, such as by assigning different net charges to the two spheres, the argument for a mirror-symmetric equilibrium no longer holds. In this unbalanced scenario, each sphere experiences a distinct local electric field, leading to an uneven distribution of induced surface charges, carrying fundamental implications for the net electrostatic force between them, as illustrated in Figure 1b. Unlike the case of two identical bodies, where the induced charges are exact reflections of one another, the charge redistribution in the unbalanced system follows a more intricate pattern. The stronger electric field of the more highly charged sphere exerts a dominant influence on its counterpart, inducing a localized region of opposite charge near the point of the closest approach. This induced charge then transitions through a neutral region before progressively adopting a charge of the same sign as its original given (free) charge on the far side. Notably, when integrated over the surface, the total charge remains unchanged; the redistribution arises from the induction of polarization (bound) charges, which locally modify the surface charge density while preserving the system’s net charge. The more highly charged sphere also undergoes polarization, though to a lesser extent. Accordingly, its surface charge density increases in magnitude near the point of the closest approach and then exhibits a local dip before stabilizing at an approximately constant value on the far side.

At sufficiently small surface-to-surface separations, *s*, where polarization effects become more pronounced, a well-established but counterintuitive phenomenon can emerge: net attraction between like-charged dielectric particles, commonly termed like-charge attraction.^39,56–63^ This occurs when localized regions of opposite induced charge form on both spheres near the point of the closest approach. Specifically, the sphere with the higher net charge polarizes its equally sized counterpart in an asymmetric fashion, inducing a strong region of opposite charge near the contact area, while its own polarization leads to a weaker, complementary redistribution. These induced charges generate an attractive force that under appropriate conditions can overcome the inherent repulsion between the free charges. Although the total free charge on each sphere remains conserved, the spatial rearrangement of bound charges gives rise to an interaction that is often well approximated by a dipolar term, though contributions from higher-order multipoles may also be significant depending on the system, particularly at short distances. The resultant force reflects a competition between long-range repulsion (dominated by free charges) and short-range attraction (driven by induced charge asymmetry), with the latter potentially prevailing at separations where polarization effects are maximized. The balance between these opposing forces is governed by both the degree of charge imbalance and the relative permittivity of the spheres. In particular, as the relative permittivity increases, the induced bound charge density is enhanced, thereby intensifying the short-range attractive interactions. Moreover, a sufficiently large charge imbalance is necessary to tip the balance in favor of attraction. Together, these factors determine the transition from a regime dominated by long-range repulsion to one where short-range, polarization-driven attraction prevails, as illustrated in Figure 1b.

Symmetry breaking also arises when the interacting spheres differ in size. As shown in Figure 1c, when all other parameters remain identical, the smaller sphere exerts a disproportionately strong polarizing influence on the larger counterpart. This asymmetry stems from the curvature dependence of surface electric fields: the smaller sphere, with its higher curvature, generates a more intense local field near the point of the closest approach. This enhanced field induces a concentrated region of the opposite bound charge on the adjacent surface of the larger sphere. Conversely, owing to its lower curvature, the larger sphere exerts a comparatively weak polarizing influence on the smaller sphere, thereby allowing the latter to retain a positive net surface charge density across its entire surface. The resulting charge asymmetry alters both the magnitude and the spatial profile of the electrostatic force. At sufficiently small separations, the localized opposite charges induced on the larger sphere can dominate over the repulsion from free charges, leading to a net attractive interaction—a geometric route to like-charge attraction. The general strength and onset of this effect are therefore critically dependent on four key factors: (i) the charge ratio, which sets the relative intensities of the free charges and thus the net field imbalance; (ii) the size ratio, which governs the disparity in curvature-driven surface fields; (iii) the dielectric contrast between media, which determines how efficiently polarization charges are induced; and (iv) the separation distance, which controls the spatial overlap of the induced charge distributions. Polarization-driven attraction can overcome the long-range repulsion from free charges especially when these factors align—i.e., a large charge imbalance, a pronounced size difference, high relative permittivities, and sufficiently small particle separations.

Figure 1e maps the electrostatic force between two spheres as their free charges vary from negative to positive values, while all other parameters—radii, relative permittivity, and surface-to-surface separation—are held fixed. As anticipated, oppositely charged configurations (second and fourth quadrants) exhibit uniformly attractive forces with the force magnitude increasing proportionally to the magnitude of the net charges. In contrast, like-charge interactions (first and third quadrants) are dominated by repulsion (light-blue regions), except for two narrow bands in each quadrant of strong charge asymmetry (light red), where polarization-induced attraction overcomes repulsion. This underscores that a strong charge imbalance is essential for generating like-charge attraction, whereas more balanced charge distributions maintain a predominantly repulsive interaction.

Similarly, Figure 1f maps the electrostatic force between two like-charged spheres as their radii span 2 orders of magnitude. Nearly identical radii (*a*_1_ ≈ *a*_2_) yield purely repulsive interactions, consistent with the symmetric case. However, an increasing size disparity triggers a reversal to an attractive regime. For example, along the horizontal line *a*_1_ = 10, reducing *a*_2_ from 10 (identical spheres) initially weakens repulsion and then induces attraction as *a*_2_ decreases further. This reversal arises from the interplay between curvature and polarization: the smaller sphere (*a*_2_ ≪ *a*_1_) generates a high surface field due to its reduced radius of curvature, strongly polarizing the larger sphere and creating a localized region of the opposite bound charge. The resulting multipoles dominate at short separations. Increasing *a*_2_ above *a*_1_ = 10 produces a similar trend—a weakening of repulsion that eventually becomes attraction—but the net attractive force is less pronounced; in this scenario, the curvature mismatch is less extreme, and the overall surface charge density decreases, thus generating a more modest polarization effect and, consequently, a weaker attractive interaction. The asymmetry in attractive strength between *a*_2_ < *a*_1_ and *a*_2_ > *a*_1_ regimes highlights the geometric sensitivity of polarization-driven interactions, where, given fixed free charges, smaller spheres exhibit a markedly enhanced capacity to induce polarization as a consequence of their stronger surface fields.

Taken together, these findings highlight that even subtle geometric or charge asymmetries can flip the usual repulsive scenario into an attractive regime. The dominance of short-range polarization over long-range free-charge repulsion emerges most clearly when one sphere is sufficiently smaller or more highly charged than the other, reinforcing that finite-size and curvature effects are not merely perturbative corrections but can fundamentally alter the interaction landscape.

### Three-Body Systems

Following analysis of interactions between two charged spheres, a third, neutral sphere is introduced to the system, and the influence of the original charged spheres on its response is characterized. The system is arranged linearly: the neutral sphere (Ω_2_) is positioned between two charged spheres (Ω_1_ and Ω_3_), so that Ω_1_–Ω_2_–Ω_3_ form a one-dimensional chain. Since Ω_2_ does not carry any free charge, its interaction with the charged spheres is solely mediated by the induction of bound charges on its surface by Ω_1_ and Ω_3_. This arrangement facilitates analysis of the relative polarizing influence exerted by each charged sphere on the neutral sphere. Specifically, if the electric field from Ω_1_ predominates, the net force on Ω_2_ is oriented toward Ω_1_ and is therefore conventionally assigned a negative sign. In contrast, should Ω_3_ exert a stronger influence, the net force on Ω_2_ will be oriented in the opposite direction and designated as positive. In what follows, the effects of the charge magnitude, size, dielectric properties, and separation distances on this force balance are examined.

Figure 2a illustrates how the net electrostatic force on Ω_2_ varies as a function of *s*_32_, the surface-to-surface separation between Ω_3_ and Ω_2_, while Ω_1_ and Ω_2_ remain fixed in position. Multiple curves are plotted, each corresponding to a distinct relative permittivity of Ω_2_. When ε_2_ equals that of the surrounding medium (i.e., ε_m_ = 1.0 for vacuum), there is no dielectric contrast at the boundary of Ω_2_, rendering it effectively nonpolarizable; in this scenario, the net force on Ω_2_ is zero at all separations. However, if ε_2_ > ε_m_, the sphere becomes polarized by the electric fields emanating from Ω_1_ and Ω_3_. At large *s*_32_, the influence of Ω_3_ on Ω_2_ becomes negligible, so that the net force acting on the latter is predominantly determined by the field of Ω_1_ and is directed toward it (negative sign). As *s*_32_ decreases, the influence of Ω_3_ intensifies until it balances that of Ω_1_, resulting in a net force of zero at the point where *s*_32_ = *s*_12_. Under this condition, both charged spheres are equidistant from Ω_2_ and, being identical, exert equal but opposite forces, thereby canceling the net force on Ω_2_. Reducing *s*_32_ still further drives the net force in the opposite (positive) direction, indicating that Ω_3_ now exerts the stronger polarizing influence on Ω_2_. These findings underscore the importance of separation distance: shorter gaps enhance local electric fields and, consequently, polarization effects. Conversely, as any sphere moves farther away, its ability to polarize the neutral sphere diminishes and the induced forces gradually subside.

**Figure 2:**
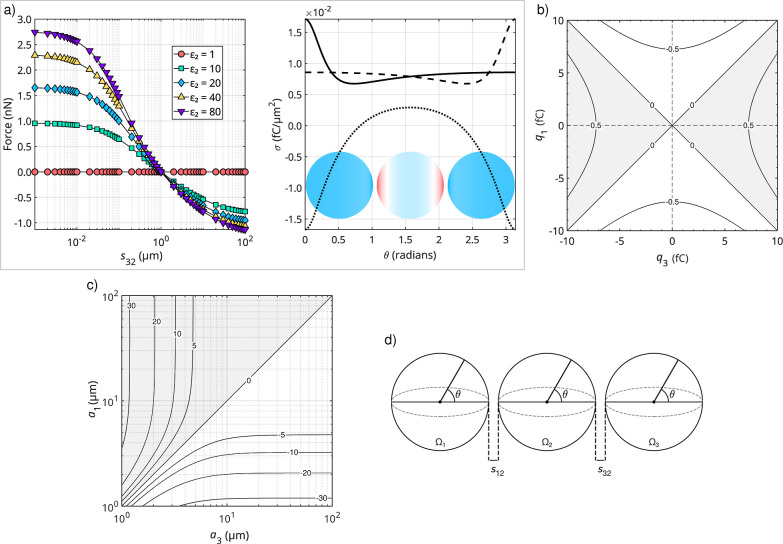
Three-sphere interactions. (a) Net electrostatic force acting on Ω_2_ due to Ω_1_ and Ω_3_, plotted as a function of the surface-to-surface separation *s*_32_ (panel on the left), and the surface charge density, σ, as a function of the polar angle, θ (panel on the right). Force curves are shown for selected values of ε_2_, as indicated, while ε_1_ and ε_3_ are fixed at 20. In all cases, Ω_2_ is electrically neutral, *q*_1_ = *q*_3_ = 10 fC, and the spheres have a fixed size of *a*_1_ = *a*_2_ = *a*_3_ = 10 μm, unless otherwise noted. The surface charge distribution is computed at *s* = 1 μm for ε_1_ = ε_2_ = ε_3_ = 20, with the solid, dotted, and dashed curves representing Ω_1_, Ω_2_, and Ω_3_, respectively. Blue hues on the spheres indicate positive charge, red hues indicate negative charge, and white denotes electrically neutral areas. (b) Contour plot of the net electrostatic force (nN) acting on Ω_2_ as a function of *q*_1_ and *q*_3_. (c) Contour plot of the net electrostatic force (nN) on Ω_2_ as a function of *a*_1_ and *a*_3_. Regions where Ω_2_ is attracted toward Ω_1_ are white, whereas those corresponding to a net force on Ω_2_ in the direction of Ω_3_ are shaded in gray. (d) Schematic of the geometry, denoting the quantities varied in the plots. In all cases, ε_m_ = 1 and *N* = 15, except for the surface charge density graph, where *N* = 35. For the contour plots, ε_1_ = ε_2_ = ε_3_ = 20 and *s* = 1 μm.

Figure 2b shows the net electrostatic force on Ω_2_ as the free charges on Ω_1_ (*q*_1_) and Ω_3_ (*q*_3_) vary from negative to positive, while all other parameters—radii, relative permittivities, and surface-to-surface separations—are held fixed. As anticipated, the sphere possessing the larger free charge produces a correspondingly stronger polarizing effect on Ω_2_, driving the net force in its direction. Contour lines corresponding to zero force highlight the balance points at which *q*_1_ = *q*_3_, reflecting equal but opposing fields from the two charged spheres. Regions where *q*_1_ > *q*_3_ (white) and *q*_3_ > *q*_1_ (gray) indicate which sphere dominates the interaction with Ω_2_. The magnitude of the net force grows with an increasing disparity between |*q*_1_| and |*q*_3_|. It is noteworthy that the contour lines exhibit symmetry, revealing that the polarizing influence remains invariant when |*q*_1_| = |*q*_3_|, irrespective of whether the charges are like-signed or opposite-signed. Under both *q*_1_ = *q*_3_ and *q*_1_ = −*q*_3_, sphere Ω_2_ lies equidistant from Ω_1_ and Ω_3_, experiencing equal and opposing forces that sum to zero. This result highlights the intrinsic symmetry of the three-sphere configuration whenever the charged spheres possess matching charge magnitudes.

Similarly, Figure 2c maps the net electrostatic force on Ω_2_ as the radii of Ω_1_ (*a*_1_) and Ω_3_ (*a*_3_) span 2 orders of magnitude, while all other parameters—relative permittivities, surface-to-surface separations, and charges—are held fixed. A diagonal contour corresponding to zero force demarcates geometrically symmetric configurations (*a*_1_ = *a*_3_). Any departure from this diagonal creates an asymmetric regime in which the sphere with the smaller radius exerts a stronger polarizing influence on Ω_2_, by virtue of its higher surface curvature. At the center of the map (*a*_1_ = *a*_3_ = 10 μm), both charged spheres match Ω_2_ in size. Moving away from this point in the first quadrant (*a*_1_ ≥ 10 μm, *a*_3_ ≥ 10 μm), the net force on Ω_2_ diminishes as the radii of Ω_1_ and Ω_3_ grow, weakening polarization effects. Conversely, in the third quadrant (*a*_1_ ≤ 10 μm, *a*_3_ ≤ 10 μm), the net force on Ω_2_ intensifies as both radii decrease, reflecting enhanced polarization by the more highly curved spheres. Focusing on the horizontal line *a*_1_ = 10 μm, when *a*_3_ = 100 μm, the net force on Ω_2_ is negative, pointing left toward Ω_1_. Since the radius of Ω_1_ is comparatively smaller than that of Ω_3_, the former generates a stronger local field, polarizing Ω_2_ more effectively. As *a*_3_ decreases from 100 μm, the net force decreases in magnitude until reaching zero at *a*_1_ = *a*_3_. Reducing *a*_3_ further reverses the force direction (now positive), pulling Ω_2_ toward Ω_3_, which has become the smaller sphere and thus the stronger polarizer; this force grows as *a*_3_ continues to decrease.

Overall, these observations illustrate how sphere size, charge, relative permittivity, and separation distance each play distinct roles in modulating the polarization interactions between dielectric particles. Even in nominally neutral bodies, the interplay of geometry and dielectric properties can alter force directions and magnitudes, underscoring the key role of induced charges in multisphere interactions.

### A Geometric Cutoff Parameter

As demonstrated by the preceding results, the significance of the curvature disparity in electrostatic interactions among charged dielectric spheres is closely linked to the surface-to-surface separation, *s*. At sufficiently small values of *s*, polarization effects become pronounced. The smaller sphere (i.e., the one with higher curvature) generates a more intense local electric field, thereby inducing a stronger dipole and higher-order multipole components in the larger sphere. This curvature-driven concentration of field lines near the smaller sphere’s surface enhances polarization-mediated interactions and frequently leads to deviations from purely Coulombic behavior. However, as the separation grows, the electric fields weaken, and the resulting polarization diminishes. Once *s* exceeds the characteristic size of the spheres by a sufficient margin, they effectively behave as point charges, rendering curvature effects negligible. In this long-range regime, the net interaction is governed primarily by monopole contributions.

Fundamentally, this crossover from “curvature-dominated” to “point-like” behavior is governed by the various length scales in the problem: the radii of the spheres (and hence their curvature) and the separation *s*. In practice, a central challenge is to identify the threshold beyond which polarization effects can be safely ignored. Therefore, to ascertain which polarization contributions to a given sphere can be neglected when all other spheres in the system are considered, the scaled surface-to-surface separation s*, introduced by Chan et al.^38^ and derived based on the bispherical coordinate system,^64^ is adopted. For each pair of spheres with radii *a*_*i*_ and *a*_*j*_, and a surface-to-surface separation *s*_*ij*_, the dimensionless quantity *s*_ij_^*^ is defined by^38^

5 $$s_{ij}^{*}=\frac{s_{ij}\left(a_{i}+a_{j}+s_{ij}\right)}{\sqrt{{\left(a_{i}+a_{j}+s_{ij}\right)}^{4}+{\left(a_{i}^{2}-a_{j}^{2}\right)}^{2}-2{\left(a_{i}+a_{j}+s_{ij}\right)}^{2}\left(a_{i}^{2}+a_{j}^{2}\right)}}$$

The parameter remains well defined for all combinations of sphere sizes and separation distances. As shown by Chan et al.,^38^ s* cleanly characterizes various geometric limits. Accordingly, s* → 1 when the gap *s*_*ij*_ is much larger than both *a*_*i*_ and *a*_*j*_ or equivalently when *a*_1_, *a*_2_ → 0 at fixed *s*, and the spheres become negligible in size relative to their separation. Consequently, curvature-induced polarization effects vanish, each charged sphere effectively behaves as a point charge, and the electrostatic force reduces to the inverse-square Coulomb’s law. In the opposite extreme, s* → 0 if *a*_1_, *a*_2_ → ∞, while *s* remains finite—or if the surfaces are so large that locally they appear nearly flat over the interaction region—the system resembles two infinite parallel planes rather than two spheres. In this regime, finite-size curvature effects are negligible not because the spheres are too small but rather because they are “infinitely large” compared to the gap. The electrostatic problem then approaches the classic scenario of planar interfaces where induced surface charges can dominate the interactions.

For typical finite-size spheres at moderate separations, s* remains of order unity O(1) , placing the system outside both the point-charge–point-charge and plane–plane limits. In this regime, curvature effects cannot, in principle, be neglected, so polarization can be significant, especially if one sphere is much smaller or more highly charged than its counterpart. Under such conditions, one must carefully account for multipole expansions or boundary-integral solutions to capture the induced charges and forces with adequate accuracy. A particularly noteworthy transition occurs at s* = 0.5, where the geometry may reflect either two finite spheres of comparable radii and separation or a point–plane interaction (*a*_*i*_ → 0, *a*_*j*_ → ∞, or vice versa). In both scenarios, curvature and polarization strongly influence the net force: two finite spheres at moderate separation deviate markedly from purely Coulombic behavior, whereas a large spherical or planar surface near a point charge experiences a pronounced polarization response. Consequently, s* ≈ 0.5 indicates a potent regime in which polarization significantly contributes to the overall interaction. These geometric scenarios, along with the limiting cases discussed previously, are illustrated in Figure 3a, where *s*_12_^*^ is plotted as a function of the surface-to-surface separation, *s*_12_, and the radius ratio of two spheres, Ω_1_ and Ω_2_.

**Figure 3:**
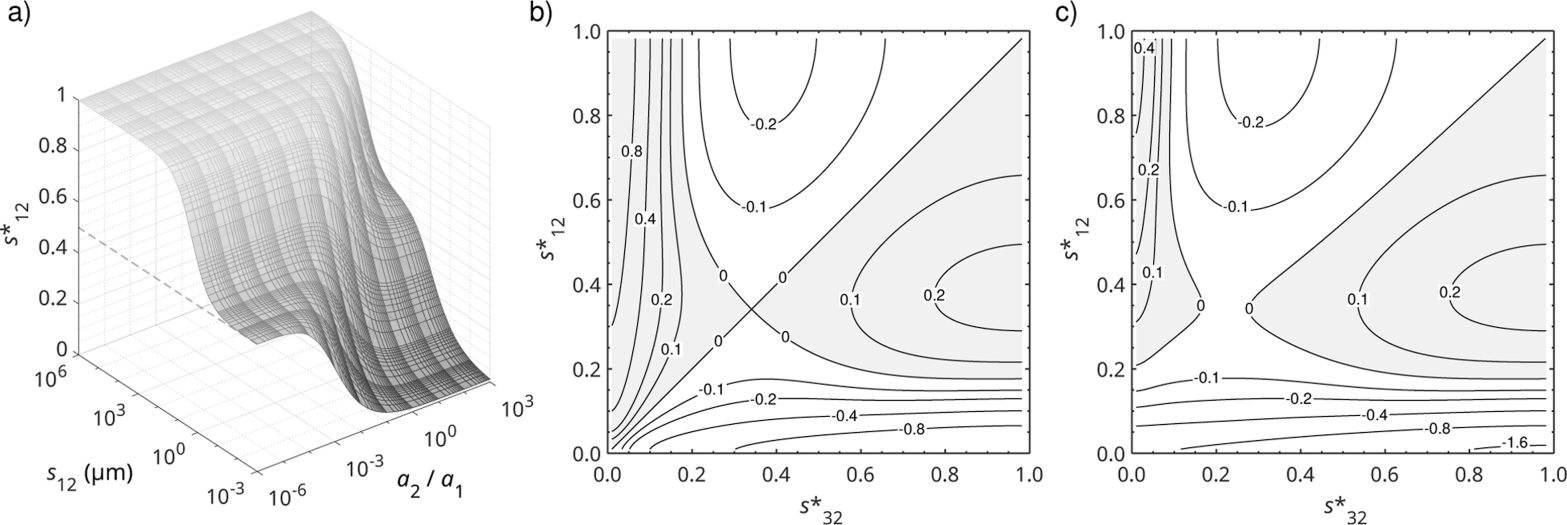
Geometric parameters. (a) Scaled surface-to-surface separation, s*, plotted as a function of the surface-to-surface separation, *s*, and the radius ratio between two spheres, Ω_1_ and Ω_2_, with *a*_1_ held at 10 μm. A dashed gray line marks s* = 0.5 to enhance visual clarity. (b) Contour plot of the net electrostatic force (nN) acting on Ω_2_ (in the same configuration shown in Figure 2) as a function of *s*_12_^*^ and *s*_32_^*^, with *a*_1_ = *a*_2_ = *a*_3_ = 10 μm, *q*_1_ = *q*_3_ = 10 fC, and *q*_2_ = 3 fC. (c) Contour plot of the net electrostatic force (nN) acting on Ω_2_ for the same system, except that *a*_3_ is set to 12 μm. In both contour plots, white regions indicate attraction of Ω_2_ toward Ω_1_, whereas gray regions denote net forces on Ω_2_ directed toward Ω_3_. For (b,c), ε_1_ = ε_2_ = ε_3_ = 20, ε_m_ = 1 and *N* = 15.

It is instructive to revisit the three-body configuration and examine how the parameter s* governs the influence of charged spheres Ω_1_ and Ω_3_ on a weakly like-charged sphere Ω_2_ (which carries the same sign of free charge but with a smaller magnitude). Figure 3b maps the net electrostatic force on Ω_2_ as *s*_12_^*^ and *s*_32_^*^ vary from near the bottom limit of 0 up to near its upper limit of 1, while the radii, relative permittivities, and net charges remain fixed. Varying the actual surface-to-surface separations *s*_12_ and *s*_32_ changes *s*_12_^*^ and *s*_32_^*^ accordingly, spanning this parameter space. A diagonal contour of zero force demarcates the symmetric geometries *s*_12_^*^ = *s*_32_^*^. Departures from this line indicate that one charged sphere lies closer to Ω_2_ than the other, thereby exerting a stronger influence. Because all three spheres are like-charged, a second, curved contour of zero force also appears, bisecting this diagonal. The region to the left of this curved zero-force contour represents conditions under which like-charge attraction emerges, whereas the region to the right corresponds to net repulsion.

In the attractive regime, s* values near 0 indicate sufficiently close separations that polarization-induced bound charges overcome the Coulombic repulsion of the free charges, aided by the asymmetry (*q*_1_ = *q*_3_ > *q*_2_). Conversely, once s* increases beyond a threshold (moving to the right of the curved zero-force contour), the Coulombic component prevails, and the system returns to the expected repulsive regime. Within the attractive region, points with *s*_12_^*^ > *s*_32_^*^ imply that the sphere Ω_3_ is closer to Ω_2_, causing a net force that pulls Ω_2_ toward Ω_3_ (gray-shaded area, positive-force contours). Points where *s*_12_^*^ < *s*_32_^*^ signify Ω_1_ is nearer to Ω_2_, and the induced attraction pulls Ω_2_ toward Ω_1_ (white-shaded area, negative-force contours). In the repulsive region, these same inequalities dictate the direction of the net force but with repulsion dominating: *s*_12_^*^ > *s*_32_^*^ pushes Ω_2_ away from Ω_3_ (white-shaded area), while *s*_12_^*^ < *s*_32_^*^ drives Ω_2_ away from Ω_1_ (gray-shaded area).

Consider, for instance, tracing a path along the line where *s*_12_^*^ = 0.6. Initially, when *s*_32_^*^ is close to zero, sphere Ω_3_ is in close proximity to Ω_2_ and, despite the free-charge repulsion, its strong polarization effect induces a net attractive force on Ω_2_ (reflected by a positive force value), while the influence of the more distant Ω_1_ is minimal. As *s*_32_^*^ increases, the system eventually crosses the curved zero-force contour; at this point, the attractive polarization forces and the repulsive Coulombic interactions between Ω_3_ and Ω_2_ are in near balance, yielding a net force close to zero (with the contribution of Ω_1_ remaining minor due to its larger separation). Beyond this initial zero-force crossing, the interaction between Ω_3_ and Ω_2_ transitions to a predominantly repulsive regime, leading to negative force values that at first increase in magnitude as Coulombic repulsion becomes dominant. However, as *s*_32_^*^ continues to increase, the corresponding separation *s*_32_ grows, progressively attenuating the Coulombic interaction. This weakening persists until the system reaches the diagonal zero-force contour, where *s*_12_^*^ = *s*_32_^*^, and complete symmetry is established. As *s*_32_^*^ continues to increase, Ω_3_ recedes further from Ω_2_, progressively diminishing its direct influence. Consequently, Ω_2_ becomes increasingly governed by interaction with Ω_1_. However, since the separation between Ω_1_ and Ω_2_ remains correspondent to *s*_12_^*^ = 0.6, their interaction remains predominantly repulsive, sustaining a net positive force value.

In Figure 3c, asymmetry is introduced by enlarging Ω_3_ relative to the other spheres (i.e., *a*_1_ = *a*_2_ < *a*_3_). The increased size of Ω_3_ results in a lower surface curvature, leading to a weaker local electric field and, consequently, a diminished capacity to induce polarization in Ω_2_. This reduction in the polarization efficiency creates an imbalance in the induced multipole contributions, shifting the force equilibrium away from the diagonal. As a result, two primary consequences emerge: first, the white-shaded region where the net force on Ω_2_ is directed toward Ω_1_ (corresponding to negative force values) becomes more extensive; and second, the symmetric bisection along the diagonal zero-force contour, characteristic of the symmetric configuration, is no longer observed.

Because s* spans the entire continuum from purely Coulombic to strongly polarization-dominated regimes, it serves as a well-defined criterion for establishing a cutoff parameter in many-body calculations. Specifically, if a given pair of spheres, Ω_*i*_ and Ω_*j*_, satisfies *s*_ij_^*^ beyond a certain threshold—say, *s*_ij_^*^ ≥ 0.9—one can argue that it can be reasonably approximated as a point-like interaction, allowing the neglect of higher-order multipole (polarization) corrections without significant loss of accuracy. Conversely, for pairs with smaller s*, one must retain the full dielectric interaction to capture the significant curvature and induction effects. This approach can drastically reduce computational overhead in large simulations, as distant pairs are effectively approximated as point–point interactions, while close pairs—where polarization matters—are treated exactly.

Real dielectric particles often depart from a perfect spherical geometry, displaying faceted rather than smooth surfaces. Sihvola’s systematic study of particle-shape effects on polarizability^65^ shows that regular polyhedra can exhibit enhanced polarizability relative to volume-equivalent spheres: icosahedra and dodecahedra show modest increases (≤10%), whereas cubes and tetrahedra show larger enhancements (+21% and +68%, respectively) due to field concentration at sharp edges and corners. Such shape-amplified polarization is also captured by the multiple-scattering theory, which demonstrates that interfacial polarization propagates through chains of neighboring particles with a strength that is highly sensitive to geometry.^66^ Although such geometric variations would rescale the polarization-induced forces computed here, the core physical mechanisms should remain qualitatively unchanged, including the geometry-based cutoff criterion and the emergence of like-charge attraction under the appropriate conditions. Future extensions to nonspherical morphologies would therefore replace the single-sphere polarizability with shape-specific values, as illustrated by recent surface-resolved simulations of charged ellipsoids.^67^

In order to solve for the electrostatic potential Φ, the model employs a second-kind integral-equation formulation combined with a spectral (spherical harmonic) discretization.^48^ Each sphere Ω_*i*_ of radius *a*_*i*_ and center *x*_*i*_ carries a free surface charge density σ_*f*,*i*_ on its boundary Γ_*i*_. The potential on Γ_*i*_ is represented by λ_*i*_. Collecting all λ_*i*_ into a single vector $\lambda ={\left(\lambda_{1},\lambda_{2},...,\lambda_{M}\right)}^{Τ}$ , and similarly all free-charge densities $\sigma_{f}={\left(\sigma_{f,1},...,\sigma_{f,M}\right)}^{Τ}$ , one obtains a linear system

6 Lλ=f

where *L* encodes the mutual polarization among all spheres, λ contains the boundary values of Φ on every sphere, and ***f*** describes the influence of the free charges.^48^ Physically, each block *L*_*ij*_ captures how induced charges on sphere *j* affect the boundary potential on sphere Ω_*i*_, ensuring a fully many-body treatment of dielectric polarization. Once eq 6 is solved for λ, one can compute the total electrostatic energy and any local fields of interest.^48^ Although the matrix *L* in principle couples every pair (*i*, *j*), many of these interactions can be considered negligible if spheres *i* and *j* lie far apart compared to their radii. To quantify this, a cutoff based on *s*_ij_^*^ ∈ [0,1] is introduced as follows: if *s*_ij_^*^ is below a user-chosen cutoff η, the corresponding off-diagonal block *L*_*ij*_ (and *L*_*ji*_) is retained in eq 6, capturing mutual polarization between spheres *i* and *j*. Otherwise, those blocks are set to zero, effectively ignoring higher-order induction between distant spheres. In this way, pairs with *s*_ij_^*^ < η remain fully coupled, while those with *s*_ij_^*^ ≥ η are treated as point-like, meaning only their free-charge interactions are computed.

From a physical standpoint, removing these distant couplings excises the part of the system where induced surface charges contribute minimally to the total energy or forces. In many contexts, large-distance interactions are dominated by monopole terms, so it is both expedient and controllably accurate to truncate beyond a certain *s*_ij_^*^. Accordingly, each sphere Ω_*i*_ retains only those matrix blocks *L*_*ij*_ for neighbors *j* lying within a distance that is small relative to the pair’s radii. This renders *L* sparse or block-sparse, cutting computational cost dramatically in large simulations while preserving short-range polarization accuracy. Choosing the cutoff value entails a transparent cost–benefit trade-off. Lower thresholds admit more pairwise blocks, improving the fidelity to intermediate-range polarization at an increased cost. Higher thresholds prune more blocks, speeding the solution yet risking underestimations of induction in borderline cases. One can fine-tune the cutoff by monitoring how energies or forces change with increasing the threshold. This ensures that the approximation remains physically reliable for the required accuracy. By calibrating this parameter appropriately, one preserves a robust geometry-informed polarization model while substantially reducing computational complexity in many-body dielectric simulations.

Figure 4 illustrates how the coupling between spheres varies as a function of the cutoff parameter η in a two-dimensional many-body system composed of spheres of differing sizes. To elucidate the results, the pairwise coupling range is quantified for two reference spheres (rendered in black): Ω_1_, one of the larger spheres, and Ω_2_, one of the smaller spheres of the aggregate. For η = 0.0, no cutoff is imposed, and both reference spheres are coupled with all others; the spheres participating in the coupling are shown in medium gray. In contrast, when η ≥ 0.9, a full cutoff is effectively enforced such that the reference spheres are decoupled from all others, with noncoupled spheres indicated in light gray. Intermediate values of η reveal distinct coupling patterns. For instance, at η = 0.1, a minor cutoff is imposed, and Ω_1_ remains coupled with nearly all other spheres. Notably, there are cases where Ω_1_ is coupled with a large sphere Ω_*i*_ at a separation *s*_1*i*_ yet is not coupled with a smaller sphere Ω_*j*_ at a separation *s*_1*j*_ even though *s*_1*i*_ > *s*_1*j*_. This observation underscores the inherently geometric nature of the cutoff, distinguishing it from standard linear criteria. Owing to its reduced size, Ω_2_ exhibits a more limited coupling range. At η = 0.2, Ω_1_ remains coupled to certain spheres—most notably to those of larger size located in the proximal region of the left band of the aggregate—whereas Ω_2_ is fully decoupled from that region. This behavior arises because, for the larger sphere, the separation to other similarly sized spheres is not sufficiently large relative to their radii to exceed the cutoff, whereas for the smaller sphere, the same absolute separation is comparatively larger. As η increases, fewer spheres remain coupled to the reference spheres. In all cases, for a given η ∈ [0,1], the coupling span for the larger sphere is consistently greater than that for the smaller sphere.

**Figure 4:**
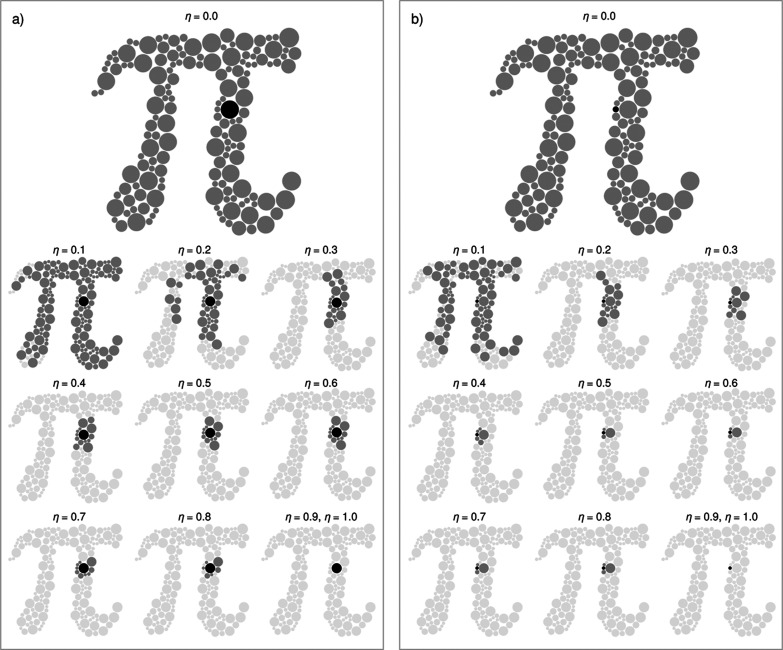
Cutoff effect. Visualization of the impact of the cutoff parameter η on coupling between reference spheres and the other spherical particles of an aggregate with heterogeneous radii, arranged on a two-dimensional surface. (a) Results for a reference sphere of radius 30.6 μm. (b) Results for a reference sphere of radius 10 μm. The reference spheres are positioned adjacent to each other to enable a clearer and more direct comparison of the effect of η. In both cases, the reference sphere is shown in black, spheres that remain coupled to the reference sphere after the cutoff is applied appear in medium gray, and those excluded by the cutoff are shown in light gray.

It is important to note that in coarse-grained or polarizable force-field models, where groups of atoms or molecules are represented as finite-sized entities, a geometry-based cutoff such as that defined via s* can offer distinct advantages by capturing when curvature-dependent polarization effects are relevant. Traditional cutoff schemes, including spherical cutoffs and reaction-field methods, are primarily designed to truncate long-range Coulombic interactions between free charges. These approaches typically employ a uniform center-to-center distance threshold, beyond which all interactions are either entirely neglected or approximated by an averaged, mean-field correction.^68^ In doing so, they do not discriminate between the finer details of polarization interactions; consequently, both the dominant monopole interactions and higher-order-induced effects are truncated uniformly. By contrast, the s*-based cutoff explicitly incorporates the radii of the interacting particles, thereby selectively retaining only those polarization couplings—namely, those arising from short-range, curvature-driven interactions—that significantly influence the many-body dielectric behavior while preserving the complete monopole contribution of the free charges. Moreover, although Ewald summation techniques (and their variants, such as Particle Mesh Ewald) offer a rigorous treatment of long-range electrostatics under periodic boundary conditions by partitioning interactions into real- and reciprocal-space contributions,^69^ they do not inherently account for the geometry-dependent aspects of polarization. Consequently, for systems in which the finite size of the particles is critical, the s*-based cutoff represents a complementary approach that more directly targets the nonadditive, short-range polarization effects than do standard distance-based or Ewald-based methods.

### Many-Body Systems

The examples presented above have focused on pairwise or three-body interactions, illustrating how geometry and charge asymmetries can overturn straightforward Coulombic expectations. In realistic applications, however, far larger configurations of charged and neutral particles frequently arise, with each particle potentially capable of inducing and sustaining complex electric fields in its neighbors. Crucially, the geometry-based cutoff parameter introduced in the previous section remains applicable in these more extensive assemblies, where a straightforward inclusion of all many-body interactions could otherwise become computationally intractable. In this section, the framework is applied to multisphere aggregates of increasing complexity, showing how careful pruning of distant polarization couplings can preserve accuracy while substantially accelerating large-scale calculations.

By imposing a cutoff in matrix *L*, certain pairwise polarization couplings are selectively removed, thereby modulating the interactions of the induced bound charges among the spheres. In a fully coupled system (i.e., in the absence of a cutoff), when sphere Ω_*i*_ is polarized by sphere Ω_*j*_, the bound charges induced on Ω_*i*_ can, in turn, polarize Ω_*j*_ as well as other spheres with which Ω_*i*_ remains coupled. These observations underscore the nonadditive, many-body nature of polarization, wherein each sphere is influenced not only by primary fields—those originating from free charges on other spheres—but also by secondary fields generated by induced bound charges.

Figure 5 illustrates this concept by considering a system composed of one charged sphere, Ω_1_ (the primary source of electric field), and three neutral spheres Ω_2_, Ω_3_, and Ω_4_, arranged linearly at increasing distances from Ω_1_ such that the gaps satisfy *s*_12_ < *s*_23_ < *s*_34_. In the absence of a cutoff (η = 0; see Figure 5a), all pairwise couplings are retained in matrix *L*. The charged sphere Ω_1_ polarizes Ω_2_, Ω_3_, and Ω_4_, and the resulting induced charges subsequently repolarize Ω_1_ (as well as one another), thereby generating a fully many-body, nonadditive cascade of polarization across all four spheres. By contrast, when η = 1 is imposed, all polarization couplings are eliminated from matrix *L* (see Figure 5e). In this configuration, Ω_1_ is the sole sphere carrying free charge but, as a consequence of the full cutoff, no induced charges develop on Ω_2_, Ω_3_, and Ω_4_. Consequently, the equipotential lines coincide with those of an isolated charged sphere in vacuum, effectively ignoring the presence of the neutral spheres. At intermediate cutoffs (0 < η < 1), only selected sphere pairs remain coupled, as determined by whether their scaled separation s* falls below the specified threshold. Consequently, distinct subsets of spheres engage in mutual polarization, thereby capturing a portion—but not the entirety—of the many-body effects observed in the fully coupled regime.

**Figure 5:**
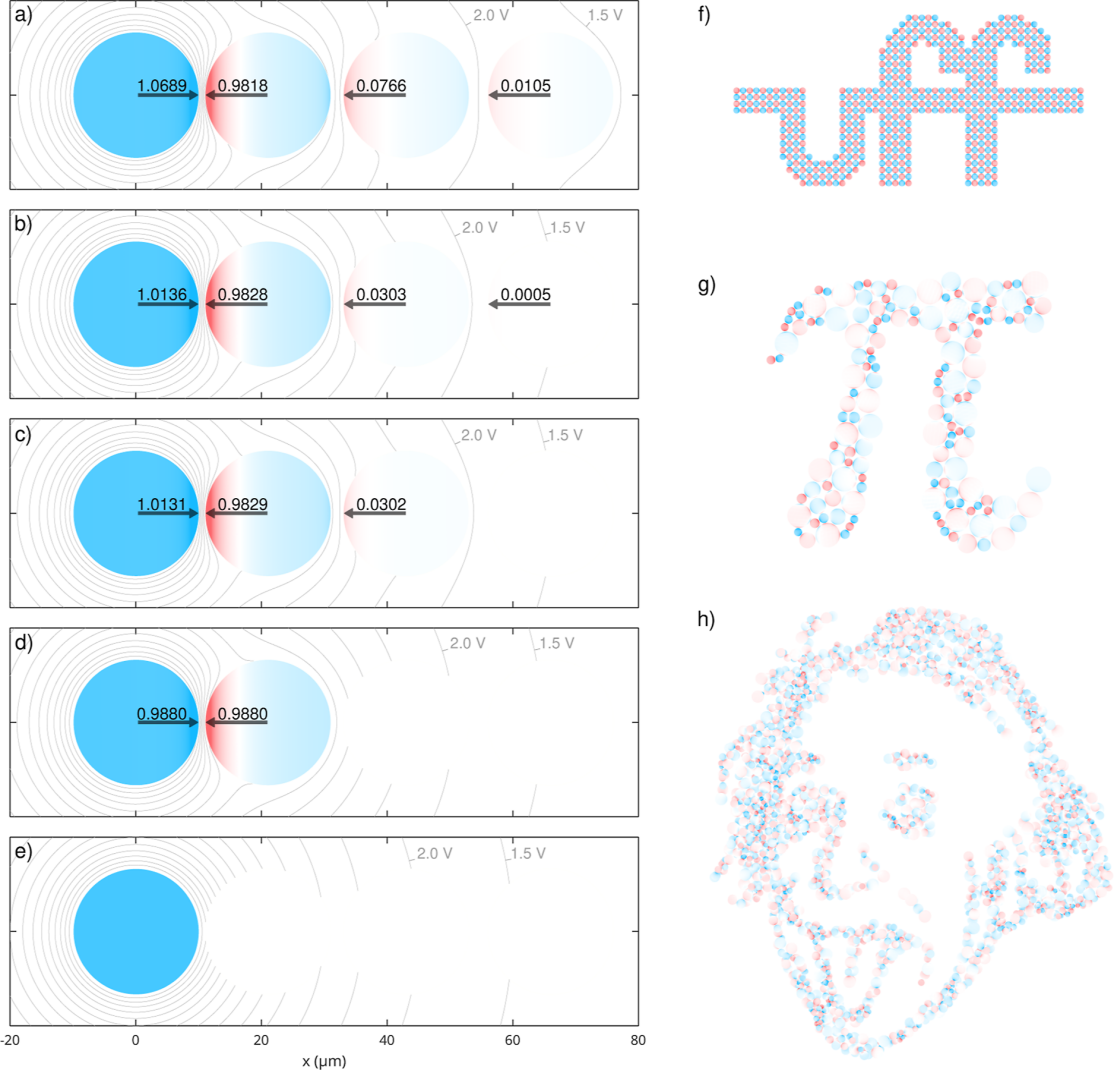
Many-sphere interactions. (a–e) System composed of four 10 μm spheres, linearly arranged (from left to right) as Ω_1_, Ω_2_, Ω_3_, and Ω_4_, with *q*_1_ = 10 fC and *q*_2_ = *q*_3_ = *q*_4_ = 0. Each panel corresponds to a different value of the cutoff parameter η, namely, (a) η = 0.00, (b) η = 0.70, (c) η = 0.75, (d) η = 0.80, and (e) η = 1.00. The surface-to-surface separation, *s*, and the scaled surface-to-surface separation, s*, among the spheres in (a–e) are reported in Table 1. Additionally, the panels display force vectors, where arrow orientations denote direction and numerical labels indicate magnitudes in nN, and equipotential contours, which are uniformly spaced at 0.5 V intervals, with the 1.5 and 2.0 V levels explicitly indicated. (f) Aggregate of 582 spheres with 10 μm, arranged on a two-dimensional grid. (g) Aggregate of 152 spheres with radii spanning 10–33.5 μm, packed on a planar surface. (h) Aggregate of 1838 spheres with radii spanning 10–30 μm, assembled into a three-dimensional cluster. All aggregates are overall electrically neutral, with individual spheres bearing alternating positive and negative charges of magnitude 10 fC. In all cases, the spheres have a relative permittivity of 20 and ε_m_ = 1. *N* = 15 for the calculations of the linear arrangement, and *N* = 10 for the aggregates. For rendering images (f,g), *N* = 18. Blue hues on the spheres indicate positive charge, red hues indicate negative charge, and white denotes electrically neutral regions.

Table 1 details both *s* and s* for each pair, thereby aiding in the interpretation of the results. For instance, at a cutoff of η = 0.80 (Figure 5d), all sphere pairs with *s*_ij_^*^ ≥ 0.20 are excluded, so that only the coupling between Ω_1_ and Ω_2_ is retained. In this configuration, Ω_2_ is polarized by Ω_1_ and polarizes it in return, while Ω_3_ and Ω_4_ remain unaffected. When the cutoff is reduced to η = 0.75 (Figure 5c), the condition is relaxed such that sphere pairs with *s*_ij_^*^ < 0.25 remain coupled. This adjustment allows the coupling between Ω_2_ and Ω_3_ to be retained, although direct coupling between Ω_1_ and Ω_3_ remains excluded since their scaled separation, *s*_13_^*^, exceeds 0.25. As a result, Ω_3_ is polarized indirectly via its interaction with Ω_2_ which is itself polarized by Ω_1_. When the cutoff is further reduced to η = 0.70, so that pairs with *s*_ij_^*^ < 0.30 remain coupled, Ω_4_ also becomes polarized. However, it remains decoupled directly from Ω_1_ and is instead polarized through the cascade Ω_1_ → Ω_2_ → Ω_3_ → Ω_4_. This configuration still does not represent the fully coupled scenario shown in Figure 5(a), as certain long-range interactions remain excluded.

**Table 1:** Pairwise Values of the Surface-to-Surface Separation, *s*, and the Scaled Surface-to-Surface Separation, s*, among the Spheres of the System Addressed in Figure 5a–e

	value of *s*				value of s*			
sphere	1	2	3	4	1	2	3	4
1		1	23	46		0.1562	0.6042	0.7314
2	1		2	25	0.1562		0.2182	0.6202
3	23	2		3	0.6042	0.2182		0.2641
4	46	25	3		0.7314	0.6202	0.2641	

Because polarization is inherently nonadditive, different cutoff scenarios yield distinct force magnitudes. In the reference, a fully coupled system (Figure 5a), the force acting on each sphere reflects the cumulative effect of induced charges from all spheres. By contrast, when only the coupling between Ω_1_ and Ω_2_ is retained (Figure 5d), the polarization state of Ω_2_ becomes exclusively governed by the electric field emanating from Ω_1_. This constrained coupling produces fundamentally divergent interparticle force profiles, with Ω_2_ experiencing an amplified force and Ω_1_ experiencing a diminished force, attributable to the absence of additional spheres mediating charge redistribution. With the introduction of additional couplings (Figure 5b–c), distant spheres such as Ω_3_ or Ω_4_ become polarized—often indirectly—thereby gradually modifying the local electric fields influencing Ω_1_ and Ω_2_. This feedback mechanism can result in an increase in certain forces (for example, by enhancing the net attractive pull on Ω_1_) while reducing others relative to the reference scenario. These subtle effects underscore the iterative, nonlinear character of polarization in many-body dielectric systems. Minor modifications in the matrix *L* can propagate through the entire ensemble, inducing shifts in bound-charge distributions and, consequently, leading to nontrivial alterations in the net forces. These examples underscore the intrinsically collective and iterative character of many-body polarization, whereby the induced charges on each sphere are determined not only by the primary electric fields generated by free charges but also by the secondary fields arising from the polarization of neighboring spheres. Consequently, even subtle modifications of the cutoff threshold may propagate throughout the system in a nonlinear manner, altering both local charge distributions and the overall force balance. This emphasizes the critical importance of carefully selecting the cutoff criterion to preserve the essential polarization effects.

To assess the computational gains and accuracy trade-offs associated with a geometry-based cutoff, three distinct sphere assemblies were examined. In the first configuration, spheres of identical size were arranged on a two-dimensional grid; in the second, spheres with varying radii were densely packed on a two-dimensional surface; and in the third configuration, spheres of different sizes were assembled into a cluster with an added third dimension perpendicular to the plane. In all cases, the spheres were assigned alternating positive and negative charges of equal magnitude, ensuring that the overall aggregate remained electrically neutral. Visual inspection of Figure 5f–h, which depicts the systems in the absence of any cutoff (η = 0), reveals that polarization-induced charge redistribution produces marked gradients in surface charge density at the sphere interfaces. These gradients manifest as localized regions of negative charge (depicted in red) and positive charge (blue), with intermediate areas exhibiting neutral interfacial domains (depicted in white). Such intricate charge distributions are not adequately captured by low-order multipole approximations (e.g., dipole or quadrupole representations), particularly in scenarios with a high number of interfacial contacts.

Introducing a cutoff η yields two immediate effects: a deviation from the fully coupled reference energy (i.e., the energy obtained with η = 0), and a substantial reduction in computational cost. As described in Table 2, even a modest cutoff (e.g., η = 0.1 or η = 0.2) significantly reduces the overall runtime, reflecting that a matrix–vector product *L* **λ** in the uncut system typically scales as $O\left(M^{2}\right)$ .^48^ Notably, the acceleration afforded by the geometry-based cutoff ultimately exceeds the performance gains achievable with a modified Fast Multipole Method (FMM), which typically attains O(M) scaling,^48^ while relatively preserving the accuracy of the fully coupled calculation. The computational advantage of the cutoff strategy arises from its explicit removal of distant pairwise polarization couplings, whereas the FMM retains all interactions and employs hierarchical multipole compression to approximate their contributions. Thus, the performance advantage of the cutoff approach is anticipated to develop and scale with the system size. This efficiency gain is attributable to the systematic decoupling of spheres whose pairwise scaled surface-to-surface separation exceeds the threshold η, thereby reducing the cardinality of non-negligible interacting pairs and conserving computational resources in proportion to the sparsity of the truncated interaction matrix. Importantly, the associated accuracy degradation appears to remain bounded—below 1% relative error for η ≤ 0.2 in the studied aggregates—since polarization interactions between far-separated spheres contribute minimally to the total polarization energy. At stricter cutoffs, error magnitudes can rise nonmonotonically due to the inherently nonadditive nature of polarization. Eliminating specific interactions may either weaken or intensify local induction, causing abrupt sign changes or large swings in the total energy. In configurations in which the truncated network isolates a sphere with only a few close neighbors, the bound-charge distribution can become more localized, inflating the remaining interactions above the fully coupled baseline. Conversely, if the pruned couplings were crucial for sustaining collective induction, then the energy could drop well below the reference.

**Table 2:** Computation Wall Time and Percentage Error in the Calculated Polarization Contribution to the Total Interaction Energy for the Aggregates Presented in Figure 5f–h, Evaluated over the Full Range of the Cutoff Parameter η^a^

	aggregate 1		aggregate 2		aggregate 3	
η	time (s)	error (%)	time (s)	error (%)	time (s)	error (%)
0.0	14117		1042		134225	
0.1	3097	–0.24	515	+0.02	16669	–0.14
0.2	912	+0.87	178	–0.50	4990	–0.28
0.3	377	+3.22	91	–0.03	2177	–1.06
0.4	242	–17.31	58	–0.60	1187	–2.83
0.5	170	–2.93	39	+2.22	754	+2.17
0.6	104	+34.36	32	+8.51	564	+4.94
0.7	104	+34.36	29	+14.85	434	+0.28
0.8	104	+34.36	26	+13.40	157	–72.37
0.9	8	–100.00	1	–100.00	78	–100.00
1.0	8	–100.00	1	–100.00	78	–100.00
FMM	864	0.00	76	0.00	7658	0.00

a The timings were carried out in serial on a standalone workstation featuring an AMD Ryzen 7 5700 CPU, paired with 32 GB DDR4-3200 RAM, running under Ubuntu 24.04.2 LTS. All routines were implemented and executed using MATLAB R2024b.^70^

In the first aggregate, Figure 5f, uniformly sized spheres are closely packed in a two-dimensional grid, giving rise to a dense network of neighbors. As η increases, the consequent removal of intermediate- and long-range interactions leads to abrupt sign changes in the energy error (ranging from −17.31% to +34.36%), reflecting a pronounced feedback loop among the numerous couplings. The second aggregate, Figure 5g, likewise exhibits a two-dimensional arrangement but includes spheres of varied radii, causing a more heterogeneous local environment. Although this system remains dense, its reduced total number of spheres and inherent size disparities serve to mitigate fluctuations in the induced-charge distributions, yielding milder deviations (under 15%) once cutoff thresholds are introduced. By contrast, the third aggregate’s, Figure 5h, three-dimensional structure contains a greater total number of spheres yet is more sparsely connected on larger length scales. Accordingly, stringent η values lead to significant reductions in the global induction energy (e.g., −72.37% when η = 0.8), but the intermediate regime exhibits more restrained swings compared to the sharply oscillatory behavior observed in the two-dimensional grids. Overall, the interplay of cutoff and local packing density—whether in uniform or size-varying spheres and in planar or volumetric arrangements—determines the extent to which removing distant couplings either amplifies local multipoles or disrupts collective induction, ultimately governing the sign and magnitude of the observed errors.

As η nears unity, induction pathways are progressively lost, and upon reaching η ≥ 0.9, all pairwise polarization couplings are removed, causing the energy to collapse to its bare Coulombic limit and resulting in a −100% relative error in polarization energy compared to the fully coupled reference. This progression highlights that polarization in finite-size dielectrics constitutes a strongly collective effect and that fine-tuning η is necessary to reconcile computational savings with the accurate representation of many-body interactions. In practical terms, s* serves as a criterion, indicating when two spheres are distant enough that their mutual influence can be approximated by point charges. Choosing a cutoff not too far above η = 0.0 retains most of the relevant polarization couplings. As discussed earlier, the limit s* = 0.5 reflects genuine sphere–sphere interactions (or, ambiguously, a point charge near a plane), so adopting a cutoff near this value would risk excluding genuinely important medium-range interactions. As suggested by the numerical results, values of η in the 0.1–0.4 range can yield acceptable trade-offs in certain contexts, but the optimal choice depends on the specific configuration and the desired accuracy. Overall, the key guideline is to avoid moving η so far from 0.0 that essential polarization effects are lost, thereby balancing computational speed with the fidelity of many-body dielectric interactions. Small η values incur only minor errors, representing negligible deviations from a physically rigorous depiction. As the value of η increases, substantial deviations from a physically realistic behavior can emerge. These errors stem from the systematic exclusion of long-range polarization couplings. Notably, the occurrence of apparently small errors at higher η values in Table 2 is fortuitous; it arises from an energetic boost contributed by low-cardinality interactions that compensate for the neglected polarization effects among more distant particles. This compensation mechanism supports the use of lower η thresholds, which preserve an essentially accurate description of the many-body polarization dynamics.

## Summary and Outlook

Polarization in dielectric systems composed of finite-size spherical particles has emerged throughout this study as a highly nonadditive phenomenon, governed not only by the primary electric fields generated by free charges but also by the feedback from induced bound charges among the interacting species. The results indicate that size effects play a crucial role whenever particles approach one another closely or differ substantially in net charge, often yielding markedly enhanced local fields and strong deviations from purely Coulombic behavior. Even nominally neutral particles become important participants in the collective response, revealing how short-range polarization can tip force balances and, in certain cases, enable attraction between like-charged species.

This work has also demonstrated that far-field interactions can be simplified considerably once separations exceed the characteristic size of the involved particles. At such distances, polarization charge induction weakens and the effective interactions approach those of point charges. Building on this observation, a cutoff parameter informed by the scaled surface-to-surface separation, s*, was introduced to distinguish regimes in which polarization must be retained from those in which particles can be approximated as monopoles. By directly encoding particle sizes along with their separation, this geometry-based criterion allows for a selective omission of multipole couplings at large distances, yielding substantial computational gains in many-body calculations. Moreover, the integral equation model^48^ employed herein has proven highly effective in capturing both short- and intermediate-range polarization effects across multiple particles, thereby ensuring stable and accurate solutions to the underlying many-body problem.

Although the geometry-based approach brings notable advantages, carefully accounting for strong correlations and closely packed clusters remains essential since even particles at moderate distances may contribute to collective induction pathways. Tuning the cutoff parameter is particularly important to avoid overlooking relevant polarization effects, and system-specific validation of the threshold helps ensure that accuracy is maintained. In addition, the theoretical framework can be extended to accommodate nonspherical morphologies, thereby broadening its predictive capacity. Future developments may integrate these enhancements, broadening the scope to more intricate particle morphologies and complex electrostatic environments.

Overall, the geometry-based approach offers a transparent vantage point on how finite size and separation collectively drive induced-charge distributions. Its ability to capture the essential short-range polarization mechanisms, while selectively pruning weak long-range contributions, provides a powerful route for accelerating large-scale simulations of dielectric particles. Equally important is its potential integration into coarse-grained or polarizable force-field models, where both systemwide dielectric screening and local-curvature-induced effects must be treated accurately. The prospects are therefore extensive, encompassing multiscale modeling in areas such as colloidal assembly, soft matter, and the design of advanced materials reliant on emergent electrostatic phenomena. By adopting this geometry-focused methodology, deeper insights can be gained into how finite-size polarization shapes interactions across length scales, paving the way for more efficient computations and more precise predictions in physical and chemical applications.

## Supplementary Material



## Notes

Enlarged versions of Figure 5f–5h, presented at a higher resolution to improve visual clarity and highlight polarization details (PDF)
