# Supplementary material for: Charge-Induced Polarization in Dielectric Particle Systems: A Geometry-Dependent Effect
Source: J Chem Theory Comput. 2025 Jun 12;21(12):6135–50. doi: 10.1021/acs.jctc.5c00544 (PMC12199467; doi:10.1021/acs.jctc.5c00544)
Supplement: Supplementary file 1 [file ct5c00544_si_001.pdf]

Supporting Information for: Charge-Induced Polarization in Dielectric Particle Systems: A Geometry-Dependent Effect

Eric B. Lindgren*[1]

[1] *Departamento de Físico-Química, Instituto de Química, Universidade Federal Fluminense, 24020-141, Niterói, Rio de Janeiro, Brazil*

May 26, 2025

High-resolution enlargements of Figures 5f, 5g and 5h from the main text are provided on the following pages.

*Corresponding author. ✉ ericlindgren@id.uff.br

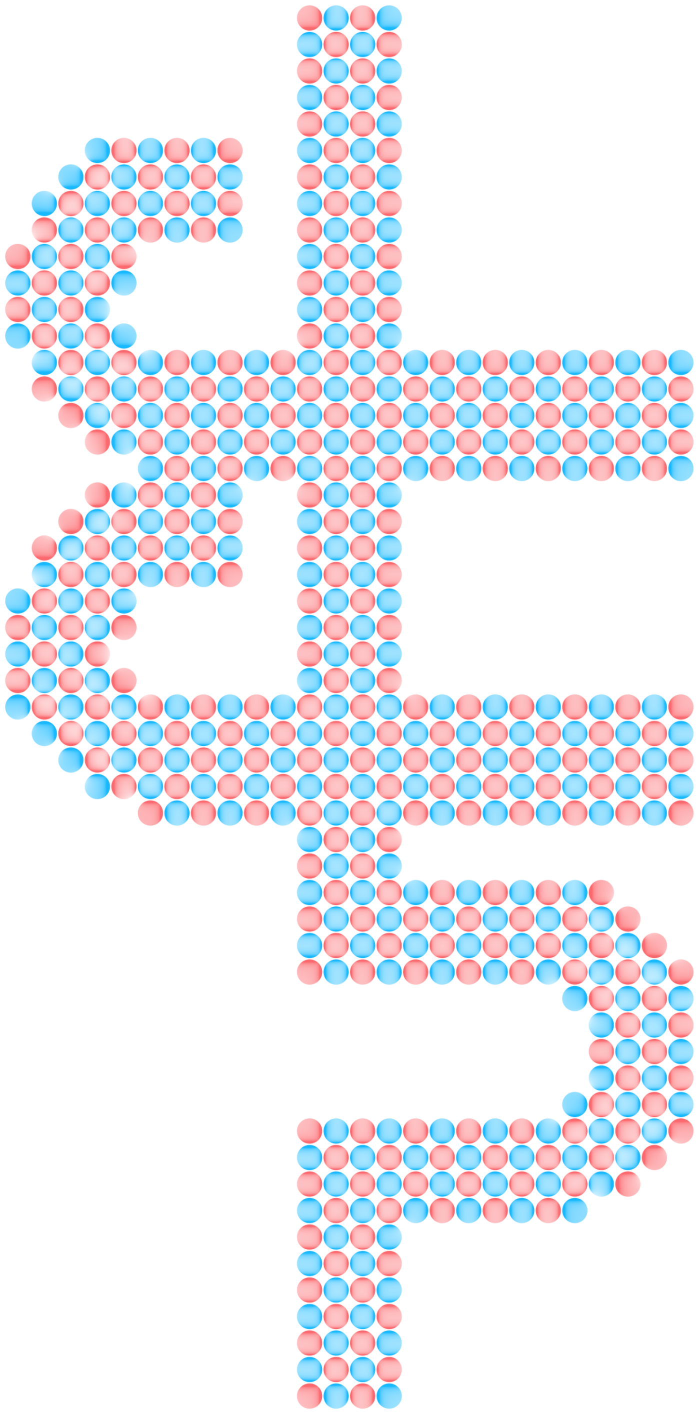

Figure S1. Enlarged view of Figure 5f in the main text.

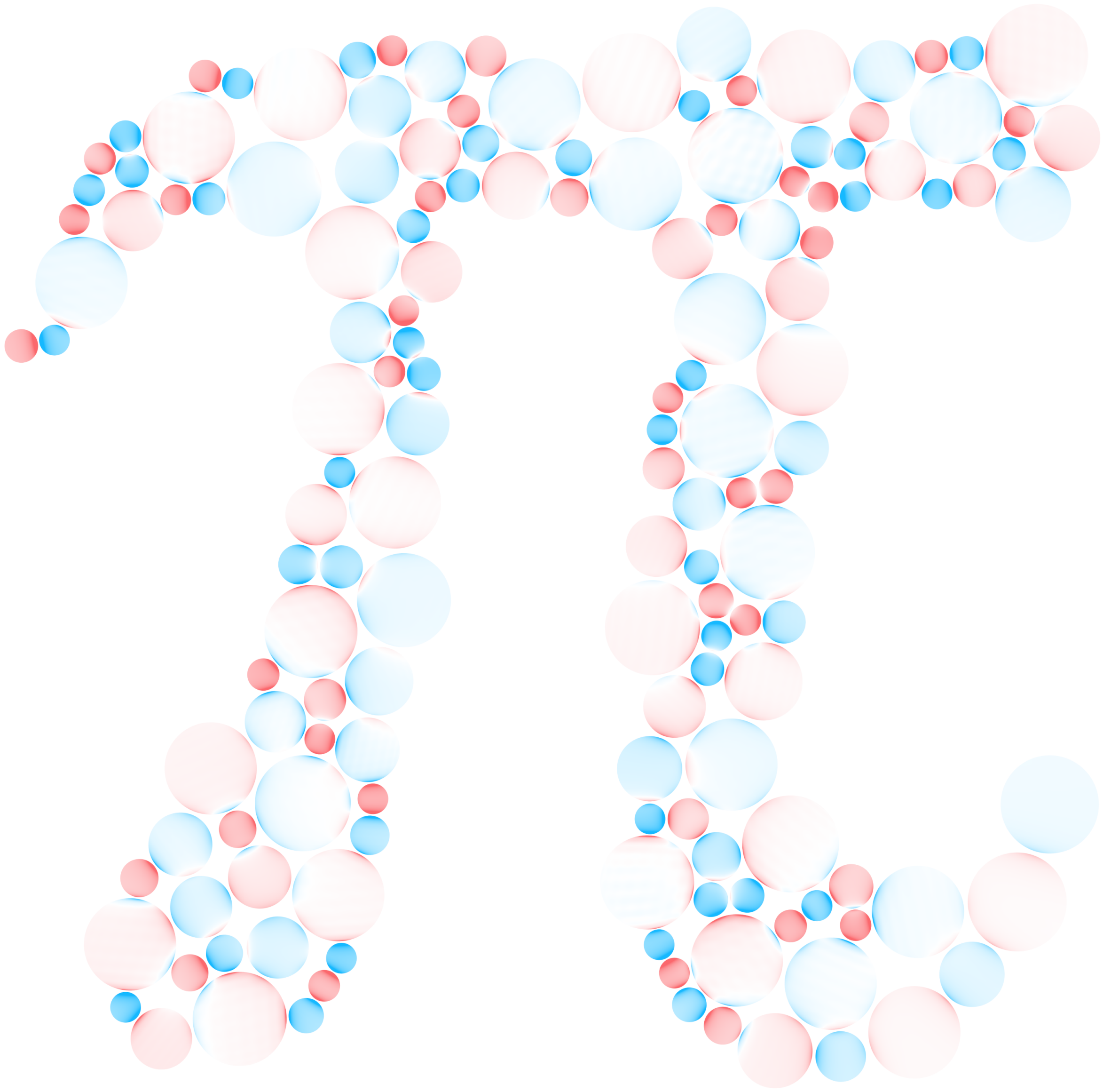

Figure S2. Enlarged view of Figure 5g in the main text.

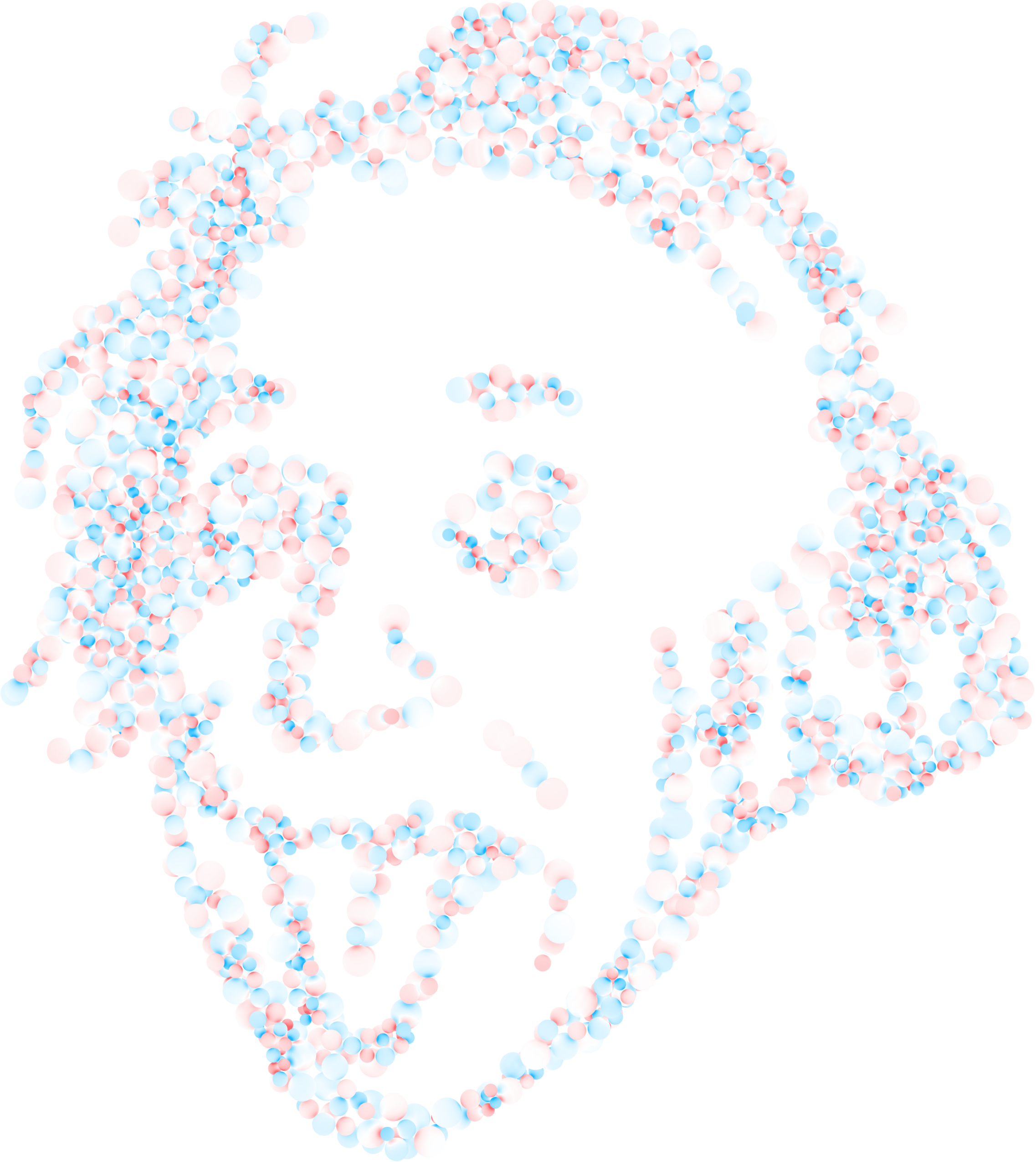

Figure S3. Enlarged view of Figure 5h in the main text.
